# Lower Circulating Trace-Element Concentrations Among SGLT2 Inhibitor Users with Diabetes: An Exploratory Cross-Sectional Study

**DOI:** 10.3390/nu18213510

**Published:** 2026-10-25

**Authors:** Beata Krasińska, Minseung Kang, Giuseppe Maria Raffa, Calogera Pisano, Mariusz Kowalewski, Siwon Baek, Piotr Suwalski, Dagmara Pietkiewicz, Szymon Plewa, Jan Matysiak, Zbigniew Krasiński, Julia Frąckowiak, Anetta Hanć, Tomasz Urbanowicz

**Affiliations:** 1 Department of Hypertensiology, Angiology, and Internal Medicine, Poznan University of Medical Sciences, ½ Dluga Street, 61-848 Poznan, Poland; 2 College of Medicine, Korea University, 73 Goryeodae-ro, Seongbuk-gu, Seoul 02841, Republic of Korea; 3 Department of Precision Medicine in Medical Surgical and Critical Area (Me.Pre.C.C.), University of Palermo, 90134 Palermo, Italy; 4 Department of Research, IRCCS ISMETT, 90127 Palermo, Italy; 5 Thoracic Research Center, Collegium Medicum, Nicolaus Copernicus University, 85-067 Bydgoszcz, Poland; 6 Department of Cardiac Surgery, Center of Postgraduate Medical Education, Central Clinical Hospital of the Ministry of Interior, 02-507 Warszawa, Poland; 7 College of Medicine, Inje University, 75 Bokji-ro, Busanjin-gu, Busan 47392, Republic of Korea; 8 Centre for Innovative Pharmaceutical Technology, Poznan University of Medical Sciences, Rokietnicka 3, 60-806 Poznan, Poland; 9 Department of Inorganic and Analytical Chemistry, Faculty of Pharmacy, Poznan University of Medical Sciences, 61-614 Poznan, Poland; 10 Department of Vascular, Endovascular Surgery, Angiology and Phlebology, Poznan University of Medical Sciences, ½ Dluga Street, 61-848 Poznan, Poland; 11 Department of Trace Analysis, Faculty of Chemistry, Adam Mickiewicz University, 61-614 Poznan, Poland; 12 Cardiac Surgery and Transplantology Department, Poznan University of Medical Sciences, ½ Długa, 61-848 Poznan, Poland

**Keywords:** sodium–glucose cotransporter-2 inhibitor, zinc, trace elements, diabetes mellitus, metabolomics, kidney function

## Abstract

Background/Objectives: Sodium–glucose cotransporter-2 inhibitors (SGLT2is) exert metabolic and renal effects extending beyond glycemic control, yet their relationship with circulating trace-element profiles remains poorly characterized. We investigated whether prevalent SGLT2i use was associated with differences in serum trace elements and targeted metabolites, with particular attention to renal function and the possibility of a shared multielement phenotype. Methods: In this exploratory cross-sectional study, serum trace elements and targeted amino acid metabolites were evaluated according to prevalent SGLT2i exposure. The principal clinical comparison comprised participants with diabetes mellitus (DM) receiving SGLT2i (*n* = 6) versus those not receiving SGLT2i (*n* = 10). Secondary analyses incorporated the broader three-group cohort. Because of the small exposed group and nonrandom treatment allocation, analyses were considered hypothesis-generating. Group comparisons were complemented by permutation testing, regression adjusted for recalculated uncapped 2021 CKD-EPI estimated glomerular filtration rate (eGFR), false-discovery-rate correction, inter-element correlation analysis, principal-component analysis, and influence diagnostics. Results: Among participants with DM, circulating Cu, Mg, Se, and Zn concentrations were lower in SGLT2i users, with concordant directions across complementary analyses. In the secondary analysis incorporating multiplicity correction across eight molecular outcomes, Cu (q = 0.015) and Se (q = 0.047) met the prespecified FDR threshold, whereas Mg and Zn did not (both q = 0.051). Exploratory principal-component analysis identified a first component explaining 70.8% of the variance across the four measured elements. PC1 was lower among SGLT2i-treated participants and remained associated with exposure after eGFR adjustment (β = −2.43, 95% CI −4.22 to −0.65; *p* = 0.008); however, this component represents statistical covariance only and should not be interpreted as evidence of a common biological mechanism or shared trace-element depletion. Conclusions: Prevalent SGLT2i use among participants with DM was associated with directionally lower circulating concentrations across several measured trace elements; however, after multiplicity correction, only Cu and Se met the prespecified FDR threshold, whereas Mg and Zn did not. The cross-sectional design, small exposed subgroup, clinical imbalance, and absence of pretreatment, dietary, urinary trace element, inflammatory, treatment duration, and adherence data preclude attribution of this pattern to SGLT2 inhibition. These findings should therefore be considered hypothesis-generating and warrant prospective validation with serial pre- and post-treatment measurements to determine whether the observed phenotype reflects pharmacological effects, treatment selection, the underlying clinical phenotype, or a combination of these factors.

## 1. Introduction

Sodium–glucose cotransporter-2 inhibitors (SGLT2is) were introduced as glucose-lowering agents for type 2 diabetes mellitus (DM), but their clinical effects extend beyond glycemic control. Large cardiovascular and kidney outcome trials have demonstrated benefits across populations with type 2 DM, chronic kidney disease, and heart failure with reduced, mildly reduced, or preserved ejection fraction [1,2,3,4,5,6,7,8,9,10]. A meta-analysis of randomized trials further supports consistent cardiovascular and kidney protection across clinically relevant subgroups [11].

These clinical observations have stimulated interest in systemic mechanisms of SGLT2 inhibition. Established pharmacologic effects include proximal tubular sodium and glucose handling, natriuresis, and tubuloglomerular feedback, whereas proposed downstream pathways include shifts in substrate utilization, ketone metabolism, oxidative stress, and inflammatory signaling [12,13,14,15]. The circulating biochemical profile observed in treated patients, however, may reflect both pharmacologic effects and nonrandom treatment selection, kidney function, diabetes severity, nutrition, and concomitant therapies.

Metabolomic studies have reported shifts in energy-substrate use and amino acid metabolism during SGLT2 inhibition [16,17]. Mineral and electrolyte studies have most consistently described modest increases in serum magnesium and changes in phosphate-regulatory pathways [18,19,20]. A previous dapagliflozin study examined zinc-α2-glycoprotein, an adipokine, rather than elemental serum zinc [21]. Direct clinical evidence relating SGLT2i exposure to measured serum zinc, copper, selenium, and magnesium concentrations remains sparse.

Zinc is required for antioxidant defense, protein structure, insulin storage and signaling, and the catalytic or structural function of numerous enzymes [22,23,24,25]. Diabetes has been associated with altered serum zinc concentrations and urinary zinc loss, although findings vary with diet, inflammation, kidney function, protein binding, and analytical methods [26,27,28,29,30,31,32]. Lower serum zinc concentrations have also been described in heart failure and adverse cardiovascular phenotypes [33,34,35]. These observations support examination of serum zinc as a methodologically sensitive exploratory biomarker; they do not support interpreting a single serum measurement as whole-body zinc status or clinical deficiency.

Carbamylation-related metabolism provides a complementary perspective. Cyanate derived from urea and myeloperoxidase-dependent reactions under inflammatory conditions can carbamylate lysine residues and generate homocitrulline [36,37,38,39]. Protein carbamylation has been associated with kidney failure, atherosclerotic risk, aging, and adverse outcomes in chronic systolic heart failure [36,37,38,39,40,41]. Kidney function is therefore an important potential confounder for both trace-element status and carbamylation-related metabolites.

We investigated serum trace-element and targeted amino acid profiles according to prevalent SGLT2i use in participants with preserved left ventricular systolic function. The principal clinical comparison was between treated and untreated participants with DM. The three-group cohort was retained as a secondary descriptive and modeling framework. Because exposure was nonrandom and only six treated participants were available, all analyses were considered exploratory and hypothesis-generating. We evaluated all four trace elements symmetrically, applied multiplicity correction across eight molecular outcomes, and examined renal-function sensitivity without interpreting the results as treatment effects.

## 2. Materials and Methods

### 2.1. Study Design, Setting, and Ethics

This observational, cross-sectional multi-omics analysis was prepared in accordance with the Strengthening the Reporting of Observational Studies in Epidemiology statement [42]. The source database contained 88 participants with paired clinical and molecular data. The analysis was restricted to participants with documented left ventricular ejection fraction (LVEF) ≥ 50%. Of the 56 eligible participants, 16 had DM (6 receiving an SGLT2i and 10 not receiving an SGLT2i), 37 had neither DM nor SGLT2i exposure, and 3 did not have DM but were receiving an SGLT2i. The DM-only cohort defined the principal clinical comparison. The 37 unexposed participants without DM were retained for secondary three-group analyses. The three exposed participants without DM were excluded from formal treatment inference and summarized descriptively.

The source cohort comprised consecutive patients referred by cardiologists for hospitalization due to stable coronary disease. All patients were electively admitted to the Internal Medicine and Hypertension Department in 2025. Concomitant food allergies, dietary supplements, and hematological or oncological disease were considered contraindications. The significant valvular pathologies diagnosed were among the exclusion criteria, as was the diagnosis or suspicion of acute coronary syndrome.

The source multi-omics cohort has been used for complementary hypothesis-driven analyses. The present study was specifically designed to evaluate SGLT2 inhibitor exposure in participants with preserved left ventricular systolic function, with trace-element status as the primary molecular domain. This analytical question, study population, exposure definition, and primary outcomes are distinct from those in separate analyses of diabetes-associated amino acid remodeling in patients with established coronary artery disease.

No formal a priori sample-size calculation was performed because the study used all eligible participants in an existing exploratory cohort. The effective sample for the principal clinical comparison was 16 participants, including only 6 SGLT2i users. The study was therefore not designed to provide a definitive estimate of an SGLT2i-specific biological effect.

### 2.2. Clinical Phenotyping and Treatment-Group Definition

Clinical variables used in the analysis included age, sex, DM status, SGLT2i use, glycated hemoglobin (HbA1c), LVEF, serum creatinine, and eGFR. LVEF was obtained from the available echocardiographic record, and only participants with documented LVEF ≥ 50% were retained. Participants were classified by DM status and concurrent SGLT2i exposure into the three groups described above.

Diabetes mellitus was diagnosed before admission by diabetologists according to current guidelines [43] and confirmed in the medical history. SGLT2i exposure was defined from the medication record at the time of sampling and therefore represented prevalent rather than incident treatment. Among the six treated participants with DM, three were receiving dapagliflozin 10 mg, and three were receiving empagliflozin 10 mg. The agents were grouped as a class because the scientific question concerned prevalent SGLT2 inhibition, and the agent-specific groups were too small for comparative inference. Medication records documented continued therapy for at least six months, but the exact treatment duration and adherence were unavailable. Individual treatment, concomitant medication, renal function, and standardized trace-element profiles of the six SGLT2i-treated participants with DM are presented descriptively in Supplementary Table S3; these individual-level data should not be interpreted as a comparison between dapagliflozin and empagliflozin.

Blood sampling and laboratory measurements were performed on admission after an overnight fast, using the same standardized sampling time point for all participants.

Serum creatinine was measured in the hospital laboratory. For the principal analyses, uncapped eGFR was recalculated for each participant from serum creatinine, age, and sex using the 2021 race-free CKD-EPI creatinine equation [44]. The clinically recorded eGFR variable, which capped values ≥ 60 mL/min/1.73 m^2^ at 60, was retained only for sensitivity comparisons; the corresponding secondary molecular estimates obtained using recorded, capped eGFR versus recalculated, uncapped eGFR are provided in Supplementary Table S1. Recalculation restored continuous renal function information above 60 mL/min/1.73 m^2^ and yielded complete, uncapped eGFR values for all 53 participants in the primary cohort.

### 2.3. Amino Acid and Related-Metabolite Measurement

Targeted amino acid analysis was performed using the MassChrom Amino Acid Analysis in Plasma/Serum LC–MS/MS kit (Chromsystems Instruments & Chemicals GmbH, Gräfelfing, Germany; 75111), including dedicated mobile phases, an analytical column, analyte-specific stable-isotope-labeled internal standards, three-level 3PLUS1 plasma calibrators, and MassCheck plasma controls.

Serum (25 µL) was mixed with 50 µL of isotopically labeled internal-standard solution and 400 µL of precipitation reagent, vortexed for 30 s, and centrifuged at 16,000× *g* for 5 min. Subsequently, 200 µL of supernatant was transferred to an autosampler vial. Calibrators and controls were processed identically to study samples.

Measurements were performed using a QTRAP 5500+ hybrid triple-quadrupole/linear ion-trap mass spectrometer (SCIEX, Framingham, MA, USA) coupled to an Agilent 1260 Infinity II liquid chromatograph (Agilent Technologies, Santa Clara, CA, USA). Chromatographic separation was performed on the dedicated MassChrom column at 25 °C using a 5 µL injection and a 20.5 min binary-gradient method. Detection was conducted in positive electrospray ionization mode using optimized multiple-reaction-monitoring transitions for each analyte and its corresponding internal standard. Data were acquired using Analyst 1.7.3 (SCIEX).

Peak integration and quantification were performed using SCIEX OS 3.3.0.12027. Concentrations were calculated from analyte-to-internal-standard peak-area ratios using 1/x-weighted linear regression against three-level plasma calibrators and expressed as µmol/L.

All samples were analyzed within a single analytical batch; therefore, no inter-batch normalization was applied. Three-level MassCheck controls were included, with the mid-level control repeated throughout the analytical sequence. Across six replicate control measurements, within-batch coefficients of variation for the 45 analytes ranged from 0.6% to 9.3% (median, 3.0%)**.** Blank injections following the highest calibrator demonstrated no quantifiable carryover.

### 2.4. Trace-Element Analysis

Samples were prepared by transferring 500 µL of serum into single-use DigiTube vessels (SCP Science Ltd., Baie-D’Urfé, QC, Canada). Subsequently, 2 mL of 65% nitric acid and 1 mL of 30% hydrogen peroxide were added. The samples were digested in a heating block system (AccuBlock Digital, Labnet, Edison, NJ, USA) at 150 °C for 4 h. After cooling, the digested material was diluted 80-fold with ultrapure water. Certified reference materials and procedural blanks were digested in the same manner as the serum samples. The final solutions were stored in polypropylene tubes at 4 °C until analysis.

Elemental concentrations of Cu, Mg, Se, and Zn were determined using an ICP-MS system (Agilent 7700×, Agilent Technologies, Tokyo, Japan) equipped with a quadrupole mass analyzer and an Octopole Reaction System (ORS). The ORS was operated in helium collision mode to reduce polyatomic interferences. Instrumental parameters were kept constant throughout the measurements: 1550 W RF power, plasma gas flow of 15 L min^−1^, nebulizer gas flow of 0.98 L min^−1^, and auxiliary gas flow of 0.9 L min^−1^. Rhodium (10 µg L^−1^) and terbium (10 µg L^−1^) were used as internal standards to correct for instrumental drift and matrix effects. Calibration curves were constructed using external calibration with matrix-matched multi-element standards (Calibration Std 3, PerkinElmer, Waltham, MA, USA) prepared in 3.25% (*v*/*v*) HNO_3_.

All calibration curves demonstrated excellent linearity, with correlation coefficients (R^2^) exceeding 0.9996. Calibration was performed using six concentration levels, ranging from 1 to 500 μg/L for Ca, Fe, and Mg, and from 0.05 to 100 μg/L for the remaining elements. Certified reference materials (CRMs), Trace Elements Serum L-1 and L-2 (Seronorm, Billingstad, Norway), were used to assess trueness and ensure traceability of the measurement results. Limits of detection (LODs) for serum samples, calculated from calibration curve parameters, were 0.011 μg/L for Cu, 0.116 μg/L for Mg, 0.267 μg/L for Se, and 0.058 μg/L for Zn. Precision was evaluated by measuring CRMs in triplicate and expressed as the coefficient of variation (CV), with values of 1.5% for Cu and 6.5% for Zn. Trueness, calculated as recovery of certified concentrations, was 96% for Cu, 94% for Zn, 98% for Mg, and 109% for Se. Student’s *t*-test confirmed no significant differences between measured concentrations (±SD) and certified values (±standard uncertainty).

### 2.5. Trace-Element Data Processing and Standardization

Following ICP–MS quantification, measured concentrations were corrected for the 80-fold dilution introduced during sample preparation and are reported throughout as serum-equivalent concentrations. The same processing procedure was applied uniformly to all samples and trace elements. Accordingly, all Cu, Mg, Se, and Zn concentrations presented in the analyses represent final serum-equivalent values, and no additional dilution correction was applied during statistical analysis.

### 2.6. Metabolite Processing and Prespecified Modules

Three prespecified biochemical modules were evaluated. The lysine/carbamylation module comprised homocitrulline, alpha-aminoadipate, pipecolate, and lysine. The one-carbon/glycine module comprised sarcosine, glycine, serine, and methionine. The transsulfuration/redox module comprised cystathionine, cystine, and cysteine sulfate. Individual metabolite values were standardized for descriptive visualization, and module scores were used as pathway-level outcomes.

Individual metabolites included in the prespecified modules were standardized across the primary LVEF ≥ 50% three-group cohort using conventional z-scores, calculated as the observed concentration minus the cohort mean divided by the cohort standard deviation. Module scores were then calculated as the arithmetic mean of the available standardized component metabolites for each participant. The one-carbon/glycine module comprised sarcosine, glycine, serine, and methionine; the lysine/carbamylation module comprised homocitrulline, alpha-aminoadipate, pipecolate, and lysine; and the transsulfuration/redox module comprised cystathionine, cystine, and cysteine sulfate.

For the four-component one-carbon/glycine and lysine/carbamylation modules, a module score was calculated when at least three component metabolites were available. For the three-component transsulfuration/redox module, at least two component metabolites were required. Module scores failing these minimum completeness criteria were assigned as missing. The resulting module scores were subsequently standardized to z-scores for regression analyses, facilitating comparison of effect estimates across molecular outcomes.

Homocitrulline, which was analyzed separately as an anchor metabolite, was log2-transformed when the measured concentration was positive and then standardized to z-scores across the analysis cohort. Non-positive or non-finite homocitrulline values were treated as missing for this analysis. Metabolite standardization was not performed when the observed standard deviation was zero; in such cases, standardized values were assigned as missing.

### 2.7. Statistical Analysis

Continuous clinical characteristics were summarized as median (interquartile range), and categorical variables as *n* (%). Standardized mean differences quantified baseline imbalance in the direct comparison between SGLT2i-treated and untreated participants with DM; Hedges’ g was additionally reported for continuous variables. Three-group descriptive comparisons used the Kruskal–Wallis test for continuous variables and Fisher’s exact test for categorical variables. These *p* values describe the cohort and were not used to establish baseline comparability.

Because the exposure question was defined within diabetes, analyses restricted to the 16 participants with DM constituted the principal clinical comparison. The three-group model was retained as a secondary analysis to describe the broader phenotype structure and to compare results with the original analytical framework. Influence was evaluated across all participants in the DM-only cohort rather than only among treated participants. Leave-one-observation-out estimates and standard regression influence measures were examined for zinc and copper.

The principal DM-only regression compared the 6 SGLT2i-treated participants with the 10 untreated participants and adjusted only for recalculated uncapped eGFR. Additional covariates were not entered simultaneously because the effective sample was too small to support extensive multivariable adjustment. As an exploratory coefficient-stability analysis, BMI, HbA1c, diuretic use, and MRA use were added individually to the eGFR-adjusted Zn model rather than entered simultaneously; these sensitivity models are reported in Supplementary Table S5.

Given the small number of treated participants, distribution-free analyses supplemented the HC3-based regressions. Exact two-sided Mann–Whitney tests, rank-biserial effect sizes, and exhaustive permutation tests were applied within the DM-only cohort. The permutation procedure evaluated all 8008 possible allocations of six exposed participants among 16 participants with DM. These analyses reduce reliance on normal-based confidence intervals but do not correct confounding or convert the cross-sectional comparison into causal evidence.

No single trace element was assigned a privileged multiplicity family. Benjamini–Hochberg false-discovery-rate correction was applied across eight molecular outcomes: Cu, Mg, Se, Zn, homocitrulline, and the three prespecified metabolic-module scores. Zinc and copper were evaluated using the same DM-only, distribution-free, and influence-analysis framework. All adjusted and unadjusted results were interpreted as exploratory.

For the principal clinical comparison, each outcome was modeled within the DM-only cohort as a function of prevalent SGLT2i use, with and without adjustment for recalculated uncapped eGFR. The SGLT2i coefficient represented the standardized difference between treated and untreated participants with DM. A secondary three-group model used DM without SGLT2i as the reference category and included indicator terms for DM with SGLT2i and no DM.

The three-group analysis does not increase the number of exposed participants and requires common-slope and residual-variance assumptions across clinically distinct groups. It was therefore treated as secondary and was not used to imply that the treatment comparison involved 53 participants.

The unadjusted DM-only model was specified as Y = β_0_ + β_1_(SGLT2i) + ε. The renal-adjusted DM-only model was specified as Y = β_0_ + β_1_(SGLT2i) + β_2_(eGFR) + ε. Secondary three-group models additionally included an indicator for participants without DM.

In the DM-only models, β_1_ represented the SGLT2i-associated standardized mean difference. HC3 heteroskedasticity-consistent standard errors and normal-based 95% confidence intervals were reported for continuity with the original analysis, but their instability in a 6-versus-10 comparison was acknowledged explicitly. Exact and permutation results were used as complementary small-sample evidence.

Two-sided *p* values were reported. Benjamini–Hochberg correction was applied across all eight molecular outcomes in the principal multiplicity analysis [45]. An FDR-adjusted q value < 0.05 was reported as a statistical threshold, but no result was treated as confirmatory because the analysis was exploratory, nonrandomized, and based on six exposed participants.

Renal-adjusted models used recalculated uncapped 2021 CKD-EPI eGFR. The clinically recorded eGFR variable, which capped values ≥ 60 mL/min/1.73 m^2^ at 60, was retained only for sensitivity comparisons. HC3 confidence intervals were calculated as the coefficient ± 1.96 × the HC3 robust standard error [46], with the small-sample limitation stated explicitly.

The principal DM-only analysis included 16 participants before outcome-specific missingness. Secondary three-group models included 53 participants. No covariates were imputed. The recorded capped-eGFR sensitivity models included 51 participants because two participants lacked the corresponding recorded renal variable.

Small-sample influence analyses provided additional context. When each of the 16 participants with diabetes was omitted sequentially, the renal-adjusted SGLT2i coefficients for both zinc and copper remained negative in every iteration. The magnitude of the estimates varied, as expected in a 6-versus-10 comparison, but no single observation reversed the direction of either association. These analyses should not be interpreted as independent replication or evidence of robustness; rather, they indicate that the observed direction of association was not generated solely by one influential participant.

Analyses were performed in R version 4.6.0 (24 April 2026; R Foundation for Statistical Computing, Vienna, Austria) on macOS Tahoe 26.5.2 (ARM64). Principal package versions were dplyr 1.2.1, tidyr 1.3.2, ggplot2 4.0.3, broom 1.0.13, sandwich 3.1.1, lmtest 0.9–40, openxlsx 4.2.8.1, readxl 1.5.0, flextable 0.9.12, and officer 0.7.5.

## 3. Results

### 3.1. Study Cohort and Baseline Imbalance

**Figure 1:**
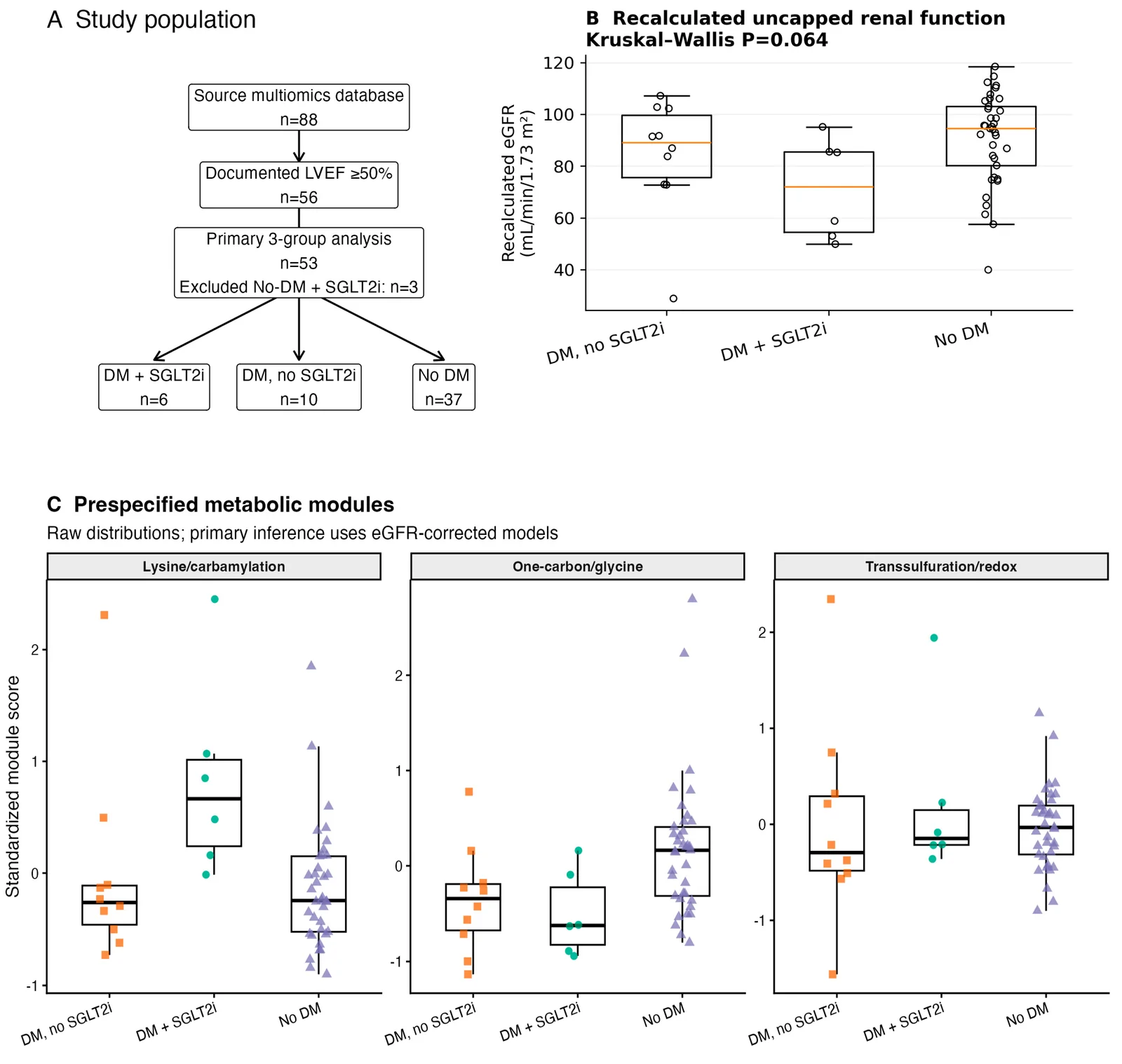
Study population, recalculated renal function, and prespecified metabolic modules. Panel (**A**) shows cohort selection, including the three SGLT2i users without DM who were retained for descriptive assessment but excluded from formal treatment inference. Panels (**B**,**C**) show secondary three-group descriptive distributions of uncapped eGFR and the prespecified metabolic modules.

The LVEF ≥ 50% cohort contained 56 participants. The principal DM-only comparison comprised 6 participants receiving an SGLT2 inhibitor and 10 participants with DM not receiving an SGLT2 inhibitor. Secondary three-group analyses included these 16 participants together with 37 participants without DM or SGLT2i exposure. Three additional SGLT2i users without DM were excluded from formal treatment inference and retained exclusively for descriptive assessment (Figure 1).

The principal baseline characteristics are summarized in Table 1, with the complete standardized imbalance assessment, including Hedges’ g for continuous variables, provided in Supplementary Table S2.

### 3.2. Trace-Element Profile in the Diabetes Cohort

All four measured trace elements were lower among SGLT2i-treated participants with DM than among untreated participants with DM. The mean between-group differences were −357.7 µg/L for Cu, −4894.4 µg/L for Mg, −30.2 µg/L for Se, and −332.3 µg/L for Zn. Because the same directional pattern extended across all four elements, subsequent analyses evaluated both individual elements and their shared covariance structure. Individual-level trace-element distributions and corresponding renal-function sensitivity analyses are shown in Supplementary Figure S1.

In exact DM-only comparisons, Cu, Se, and Zn differed between treated and untreated participants (exact Mann–Whitney *p* = 0.016, *p* = 0.022, and *p* = 0.031, respectively), whereas the corresponding result for Mg was less pronounced (*p* = 0.118). Rank-biserial effect estimates were −0.733 for Cu, −0.700 for Se, −0.667 for Zn, and −0.500 for Mg. Exhaustive permutation analyses, evaluating all 8008 possible allocations of six exposed participants among the 16 participants with DM, yielded *p* = 0.014 for Cu, *p* = 0.042 for Mg, *p* = 0.013 for Se, and *p* = 0.038 for Zn (Table 2). Detailed element-specific descriptive, distribution-free, permutation, and renal-adjusted estimates are provided in Supplementary Table S4.

### 3.3. Exploratory Inter-Element Correlations and Principal-Component Analysis

The concordant direction of the four trace elements prompted an exploratory assessment of their shared structure. All six pairwise Spearman correlations were positive, ranging from ρ = 0.512 for Se–Zn to ρ = 0.623 for Cu–Mg (all *p* < 0.001). The corresponding correlations were ρ = 0.577 for Cu–Se, ρ = 0.527 for Cu–Zn, ρ = 0.598 for Mg–Se, and ρ = 0.556 for Mg–Zn.

As an exploratory dimensional-reduction analysis, PCA was used to summarize covariance among the four measured trace elements rather than to define a biological trace-element phenotype. PC1 explained 70.8% of the total variance, with positive loadings for Cu (0.876), Mg (0.917), Se (0.870), and Zn (0.685). Thus, PC1 provides a statistical summary of their correlated circulating concentrations. Importantly, PCA cannot determine why these elements covary and cannot distinguish potential contributions from nutritional status, inflammation, protein binding, renal handling, volume status, treatment selection, or preanalytical factors. PC1 was therefore considered strictly exploratory and was not treated as a confirmatory endpoint or evidence of a common biological mechanism. Individual trace-element distributions, pairwise inter-element correlations, and PC1 are visualized in Supplementary Figure S4.

Within the DM-only cohort, mean PC1 was −2.10 among SGLT2i users compared with 0.18 among untreated participants (exact Mann–Whitney *p* = 0.022). After adjustment for uncapped eGFR, SGLT2i exposure remained associated with a lower PC1 score (β = −2.43, 95% CI −4.22 to −0.65; *p* = 0.008). These findings should be interpreted solely as evidence of statistical covariance within the present dataset and not as evidence of a shared biological mechanism.

### 3.4. Eight-Outcome Multiplicity Analysis

The secondary three-group analysis provided a complementary assessment of the molecular profile after adjustment for recalculated uncapped eGFR. Following Benjamini–Hochberg correction across all eight molecular outcomes, Cu remained associated with SGLT2i exposure (β = −1.52, 95% CI −2.48 to −0.56; *p* = 0.002; q = 0.015), as did Se (β = −1.15, 95% CI −2.04 to −0.25; *p* = 0.012; q = 0.047). Mg (β = −1.33; *p* = 0.024; q = 0.051) and Zn (β = −0.73; *p* = 0.026; q = 0.051) did not meet the prespecified FDR threshold.

Neither homocitrulline nor any of the three prespecified metabolic-module scores was supported after renal adjustment and eight-outcome multiplicity correction. The descriptive homocitrulline phenotype and the sensitivity of the original association with renal-function specification are shown in Supplementary Figure S2. Accordingly, the secondary analysis did not identify zinc as an isolated molecular signal; rather, the strongest multiplicity-adjusted associations occurred within the broader trace-element domain (Table 3).

### 3.5. Influence Analyses of Zinc and Copper

Because the principal comparison contained only 16 participants with DM, leave-one-observation-out analyses were performed across all participants, rather than only across the six exposed individuals.

For Zn, the full renal-adjusted coefficient was −0.78 SD. Across the 16 leave-one-observation-out models, the SGLT2i-associated coefficient ranged from −1.01 to −0.68 SD and remained negative in every iteration. The maximum Cook’s distance was 0.251, and the maximum absolute DFBETA for the exposure coefficient was 0.744.

For Cu, the full renal-adjusted coefficient was −1.44 SD, with leave-one-observation-out estimates ranging from −1.72 to −1.31 SD. The direction likewise remained negative in all 16 iterations. The maximum Cook’s distance was 0.498, and the maximum absolute exposure DFBETA was 0.547.

These analyses indicate that the direction of the Zn and Cu coefficients was not attributable to the omission or inclusion of a single participant. They do not, however, establish robustness, reproducibility, or protection from confounding in this small observational sample. Complete symmetric influence diagnostics for Zn and Cu are summarized in Supplementary Table S6.

### 3.6. Descriptive Findings in SGLT2i Users Without Diabetes and by Individual Agent

The three SGLT2i-treated participants without DM were examined descriptively and were not included in formal treatment inference. Their individual serum-equivalent Cu concentrations ranged from 899.6 to 1298.3 µg/L, Mg from 20,872.8 to 22,379.1 µg/L, Se from 87.1 to 132.1 µg/L, and Zn from 1085.5 to 1255.7 µg/L. Thus, these three observations did not reproduce the uniformly lower trace-element profile observed among SGLT2i-treated participants with DM. Given the sample size of three, no statistical comparison or inference regarding modification by diabetes status was performed. Their individual serum trace-element concentrations and observed ranges are reported in Supplementary Table S7.

Among the six SGLT2i-treated participants with DM, three received dapagliflozin, and three received empagliflozin. Individual trace-element profiles showed substantial within- and between-agent variation. Because each agent subgroup contained only three participants, these observations were displayed descriptively without hypothesis testing and do not permit inference regarding agent-specific effects. Agent-specific trace-element distributions are shown descriptively in Supplementary Figure S5.

### 3.7. Integrated Exploratory Interpretation of the Molecular Findings

The revised analyses identified concordant directional differences across the four measured trace elements, although the strength of statistical support differed by element. Cu and Se met the prespecified FDR threshold in the secondary eight-outcome analysis, whereas Mg and Zn did not (q = 0.051 for both). The positive inter-element correlations and a common principal component summarize the covariance within the dataset but do not establish a coordinated biological phenotype or a common mechanism.

These observations remain exploratory. The analyses derive from the same small, clinically imbalanced dataset and therefore represent complementary characterization of a single signal rather than independent confirmation. They cannot distinguish SGLT2i exposure from treatment selection, diabetes severity, renal or cardiovascular phenotype, nutritional or inflammatory factors, volume status, or concomitant medication use.

## 4. Discussion

In this exploratory cross-sectional study, Cu, Mg, Se, and Zn concentrations were directionally lower among SGLT2i-treated participants with diabetes. However, after correction for multiple testing across eight molecular outcomes, only Cu and Se met the prespecified FDR threshold (q = 0.015 and q = 0.047, respectively), whereas Mg and Zn did not (q = 0.051 for both). Positive inter-element correlations and exploratory PCA demonstrated covariance among the four circulating measurements, but these analyses provide a statistical description of the dataset rather than evidence of a coordinated biological process. In particular, PC1 cannot distinguish shared biological regulation from nutritional status, inflammation, protein binding, renal handling, volume status, treatment selection, or preanalytical influences. The multielement findings should therefore be interpreted as hypothesis-generating evidence of correlated variation in circulating trace elements rather than as a demonstration of a unified trace-element phenotype or treatment-induced depletion.

SGLT2 inhibitors modify proximal tubular sodium and glucose handling, natriuresis, and tubuloglomerular feedback [12,13,14,15], providing a biologically plausible context in which circulating mineral concentrations might vary. The present study, however, did not assess urinary excretion, intracellular or tissue distribution, dietary intake, or longitudinal changes in trace-element concentrations. Consequently, the observed profile cannot be assigned to altered renal handling or to SGLT2 inhibition itself. Differences in nutritional status, inflammation, volume distribution, protein binding, concomitant pharmacotherapy, or the clinical characteristics that influenced treatment selection remain equally plausible explanations. The present observations should be regarded as an exploratory pattern of circulating biochemical differences that require prospective mechanistic validation, rather than as evidence of treatment-induced trace-element depletion.

Zinc contributes to antioxidant defense, protein structure, intracellular signaling, insulin biology, and metalloenzyme function [22,23,24,25]. Prior SGLT2i research has addressed magnesium, phosphate, and zinc-α2-glycoprotein, but zinc-α2-glycoprotein is an adipokine and is not a measure of elemental serum zinc [18,19,20,21]. Serum zinc is influenced by dietary intake, inflammatory state, renal handling, albumin and other binding proteins, sampling conditions, and analytical methodology [24,25]. Accordingly, the lower circulating concentration observed here should not be interpreted as evidence of zinc deficiency or reduced whole-body zinc stores. More importantly, the parallel behavior of copper, selenium, and magnesium argues against interpreting zinc in isolation and places the zinc finding within a broader biochemical phenotype.

The multivariate analysis provides an exploratory summary of covariance among the measured elements. Moderate positive correlations and concordant PC1 loadings indicate that Cu, Mg, Se, and Zn varied together within this dataset, but neither correlation nor PCA identifies the source of that covariance. Accordingly, the lower PC1 score among SGLT2i-treated participants should not be interpreted as evidence of a common biological pathway, nutritional deficiency, altered renal handling, or an SGLT2i-specific effect. Importantly, the multiplicity-adjusted element-specific results were not equivalent: Cu and Se met the prespecified FDR threshold, whereas Mg and Zn did not.

Renal function warrants particular caution in interpreting these observations. Recalculation of uncapped eGFR eliminated the information loss caused by right-censoring values above 60 mL/min/1.73 m^2^, thereby strengthening the renal sensitivity analysis. It did not, however, eliminate renal confounding. eGFR does not capture tubular handling of individual elements, urinary mineral losses, or treatment-related changes in intravascular volume and filtration dynamics. Moreover, with only six exposed participants, multivariable modeling cannot reliably disentangle these interrelated processes. Persistence of the associations after eGFR adjustment should therefore be interpreted as evidence that the observed pattern is not explained by the recorded filtration estimate alone, rather than as proof of renal independence or a direct pharmacological effect.

The nonrandom nature of treatment exposure represents an equally important source of uncertainty. SGLT2i therapy was prevalent rather than newly initiated, and pretreatment trace-element concentrations were unavailable. The treated and untreated diabetes groups also differed in several metabolic, renal, and therapeutic characteristics, including marked imbalances in MRA and diuretic use. These differences raise a substantial possibility of confounding by indication and treatment-selection bias: the biochemical phenotype observed in SGLT2i users may partly characterize the patients selected for treatment rather than the biological consequences of treatment itself. The cross-sectional design cannot distinguish between these possibilities.

In contrast to the trace-element domain, the targeted metabolomic analyses did not identify a comparably coherent signal. Neither homocitrulline nor the lysine/carbamylation, one-carbon/glycine, or transsulfuration/redox modules remained supported after renal adjustment and correction across the eight molecular outcomes. This divergence is informative: within the limits of the present dataset, the principal biochemical pattern associated with prevalent SGLT2i exposure resided in the trace-element domain rather than in the prespecified amino acid pathways. The data provide no basis to propose a mechanistic coupling between the trace-element phenotype and carbamylation-related metabolism.

Several features strengthen the dataset’s internal characterization. Trace elements and targeted metabolites were measured in the same participants; renal function was reconstructed using uncapped eGFR; baseline imbalance was explicitly quantified; and the trace-element findings were interrogated using complementary exact, permutation, regression, multivariate, and influence-based approaches. The concordant direction of the four elemental signals and the preservation of the zinc and copper coefficient directions throughout leave-one-observation-out analyses further reduce the likelihood that the overall pattern arose solely from a single influential observation. These methodological features improve the credibility of the observed within-cohort pattern, but they do not overcome the limitations imposed by the small exposed group, nonrandom treatment allocation, and residual confounding.

The study has substantial limitations that define the boundaries of interpretation. Foremost, only six participants with diabetes were receiving an SGLT2 inhibitor, and the treated and untreated groups were clinically imbalanced across renal, metabolic, cardiovascular, and pharmacological characteristics. Exposure was prevalent and nonrandom; pretreatment measurements, precise treatment duration, and adherence were unavailable, precluding assessment of within-person change or temporal sequence. Dietary intake, urinary trace-element excretion, sufficiently complete inflammatory-marker data, diabetes duration, key glucose-lowering therapies, albumin and other protein-binding determinants, and detailed preanalytical variables were also unavailable. These missing determinants are particularly important for the interpretation of the PCA because covariance among circulating elemental concentrations cannot distinguish a shared biological process from common influences on intake, distribution, binding, renal handling, intravascular concentration, or sample acquisition and processing. The measurements characterize circulating concentrations and cannot establish whole-body mineral status, clinical deficiency, tissue redistribution, or renal loss. Finally, the exact tests, permutation analyses, regression models, principal component analysis, and influence diagnostics interrogate the same small dataset from complementary perspectives; their concordance strengthens internal characterization but does not constitute independent replication. External validation in an independent cohort is therefore essential.

The three SGLT2i-treated participants without diabetes provide a limited but potentially informative descriptive counterpoint. Their individual trace-element profiles did not reproduce the uniformly lower pattern observed in the treated diabetes subgroup. With only three such participants, this observation cannot support formal comparison or inference about effect modification. Nevertheless, the lack of an obviously concordant pattern outside the diabetes subgroup provides an additional reason to avoid attributing the observed phenotype directly to SGLT2 inhibition and emphasizes the potential contribution of the underlying clinical and metabolic context in which treatment was prescribed.

The appropriate next step is therefore prospective rather than further subdivision of the present dataset. Future studies should obtain trace-element measurements before SGLT2i initiation and at prespecified intervals thereafter, together with matched or propensity-balanced untreated comparators. Concurrent assessment of dietary intake, urinary elemental excretion, albumin and other binding proteins, inflammatory markers, renal tubular indices, volume status, and relevant concomitant therapies would help distinguish altered intake, redistribution, renal handling, and treatment-selection effects. Replication of the multielement pattern—and of the common trace-element component—in an independent cohort should precede any attempt to define agent-specific differences between dapagliflozin and empagliflozin or to infer clinical consequences.

## 5. Conclusions

In this small, clinically imbalanced cross-sectional cohort, prevalent SGLT2i use among participants with diabetes was associated with lower circulating concentrations of several measured trace elements. After correction for multiple testing, Cu and Se met the prespecified FDR threshold, whereas Mg and Zn did not. Exploratory inter-element correlations and PCA demonstrated covariance among the four measurements but cannot establish a coordinated biological phenotype or identify its underlying cause. The findings remain descriptive and hypothesis-generating and do not establish trace-element deficiency, treatment-induced depletion, or a causal effect of SGLT2 inhibition. Prospective studies incorporating pretreatment measurements, dietary assessment, urinary elemental excretion, inflammatory markers, albumin and other binding proteins, volume status, and standardized longitudinal sampling are required to determine whether these observations reflect pharmacological effects, treatment selection, underlying clinical characteristics, or other shared determinants.

## Figures and Tables

**Table 1:** Demographic and clinical characteristics, laboratory results, pharmacotherapy, and left ventricular performance across the three phenotype groups. The SMD column compares DM + SGLT2i with DM without SGLT2i.

Characteristic	DM + SGLT2i *n* = 6	DM, No SGLT2i *n* = 10	No DM, No SGLT2i *n* = 37	Overall *p*	SMD
Demographics					
Age, years (median (Q1–Q3))	74.00 (56.00–75.00)	65.50 (64.00–74.00)	66.00 (56.00–69.00)	0.274	0.19
Male sex (male) (*n* (%))	3 (50.0%)	3 (30.0%)	20 (54.1%)	0.426	0.42
BMI, kg/m^2^ (median (Q1–Q3))	30.81 (30.08–33.60)	27.55 (25.76–32.77)	25.40 (24.00–29.91)	0.033	0.73
Co-morbidities					
Hypertension (*n* (%))	5 (83.3%)	9 (90.0%)	27 (73.0%)	0.672	−0.20
Dyslipidemia (*n* (%))	4 (66.7%)	9 (90.0%)	32 (86.5%)	0.476	−0.59
Coronary artery disease/IHD (*n* (%))	6 (100.0%)	9 (90.0%)	29 (78.4%)	0.614	0.47
Previous myocardial infarction (*n* (%))	0 (0.0%)	1 (10.0%)	1 (2.7%)	0.517	−0.47
Atrial fibrillation (*n* (%))	1 (16.7%)	1 (10.0%)	2 (5.4%)	0.350	0.20
Chronic kidney disease (*n* (%))	1 (16.7%)	1 (10.0%)	0 (0.0%)	0.087	0.20
Laboratory results					
Creatinine, mg/dL (median (Q1–Q3))	1.02 (0.96–1.08)	0.77 (0.71–0.91)	0.80 (0.69–0.95)	0.088	0.52
Recalculated uncapped eGFR, mL/min/1.73 m^2^ (median (Q1–Q3))	72.0 (54.4–85.5)	89.2 (75.6–99.6)	94.5 (80.2–103.0)	0.064	−0.59
HbA1c, % (median (Q1–Q3))	7.50 (6.40–7.50)	6.20 (5.80–6.70)	5.60 (5.50–6.00)	0.018	0.81
Glucose, mg/dL (median (Q1–Q3))	133.50 (99.00–196.50)	111.00 (103.50–120.75)	96.00 (87.00–102.00)	0.002	0.80
Total cholesterol, mg/dL (median (Q1–Q3))	150.00 (135.00–150.00)	177.50 (170.75–202.00)	168.00 (143.00–214.00)	0.212	−1.12
LDL cholesterol, mg/dL (median (Q1–Q3))	73.00 (54.75–85.25)	114.50 (92.75–120.50)	92.00 (68.00–126.00)	0.138	−1.22
HDL cholesterol, mg/dL (median (Q1–Q3))	41.50 (39.00–46.25)	45.00 (42.25–59.00)	59.00 (47.00–69.00)	0.012	−0.61
Triglycerides, mg/dL (median (Q1–Q3))	151.00 (123.25–186.25)	113.00 (96.50–142.75)	83.00 (71.00–108.00)	0.001	0.53
Pharmacotherapy					
ACE inhibitor (*n* (%))	1 (16.7%)	7 (70.0%)	16 (43.2%)	0.117	−1.28
ARB (*n* (%))	3 (50.0%)	2 (20.0%)	10 (27.0%)	0.496	0.66
Beta-blocker (*n* (%))	3 (50.0%)	9 (90.0%)	24 (64.9%)	0.221	−0.97
MRA (*n* (%))	4 (66.7%)	1 (10.0%)	2 (5.4%)	0.002	1.43
Diuretic (*n* (%))	5 (83.3%)	3 (30.0%)	5 (13.5%)	0.002	1.28
Statin (*n* (%))	5 (83.3%)	9 (90.0%)	34 (91.9%)	0.770	−0.20
Left ventricular performance					
LVEF, % (median (Q1–Q3))	59.00 (55.75–60.00)	57.00 (54.25–60.75)	59.00 (55.00–60.00)	0.908	0.10

Values are median (interquartile range) or *n* (%). SMD denotes the standardized mean difference for the direct comparison between the two diabetic groups; the sign reflects the DM + SGLT2i minus DM without SGLT2i direction. Abbreviations: DM, diabetes mellitus; eGFR, estimated glomerular filtration rate; HbA1c, glycated hemoglobin; LVEF, left ventricular ejection fraction; IHD, ischemic heart disease; ACE, angiotensin-converting enzyme; ARB, angiotensin receptor blocker; MRA, mineralocorticoid receptor antagonist; SGLT2i, sodium–glucose cotransporter-2 inhibitor; SMD, standardized mean difference. CRP/hs-CRP was not displayed because measurements were insufficient across groups. Information on diabetes duration, metformin, insulin, and GLP-1 receptor agonist use was not available in the current analysis dataset.

**Table 2:** Small-sample evaluation of serum trace-element concentrations in the DM-only cohort.

Element	SGLT2i (*n*)	No SGLT2i (*n*)	Mean Difference, µg/L	Exact Mann–Whitney *p*	Rank-Biserial Effect	Exhaustive Permutation *p*	eGFR-Adjusted β, SD	95% CI	*p*
Cu	6	10	−357.7	0.016	−0.733	0.014	−1.44	−2.36 to −0.53	0.002
Mg	6	10	−4894.4	0.118	−0.500	0.042	−1.34	−2.65 to −0.03	0.045
Se	6	10	−30.2	0.022	−0.700	0.013	−1.23	−2.20 to −0.26	0.013
Zn	6	10	−332.3	0.031	−0.667	0.038	−0.78	−1.44 to −0.13	0.019

Values represent the principal comparison between participants with DM receiving versus not receiving an SGLT2 inhibitor. Mean differences are expressed as SGLT2i minus no SGLT2i using serum-equivalent concentrations. Rank-biserial effects are oriented in the same direction; negative values indicate lower concentrations among SGLT2i users. Exhaustive permutation tests evaluated all 8008 possible allocations of six exposed participants among 16 participants with DM. Renal-adjusted coefficients represent standardized differences after adjustment for recalculated uncapped 2021 CKD-EPI eGFR and were estimated with HC3 heteroskedasticity-consistent standard errors. Exact, permutation, and regression analyses are complementary analyses of the same observations and should not be interpreted as independent replication. Cu, copper; DM, diabetes mellitus; eGFR, estimated glomerular filtration rate; Mg, magnesium; Se, selenium; SGLT2i, sodium–glucose cotransporter-2 inhibitor; Zn, zinc.

**Table 3:** Secondary three-group molecular estimates after adjustment for recalculated uncapped 2021 CKD-EPI eGFR.

Outcome	*n*	Standardized β	95% CI	*p*	BH-FDR q
Cu	53	−1.52	−2.48 to −0.56	0.002	0.015
Se	53	−1.15	−2.04 to −0.25	0.012	0.047
Mg	53	−1.33	−2.49 to −0.18	0.024	0.051
Zn	53	−0.73	−1.37 to −0.09	0.026	0.051
Lysine/carbamylation module	53	0.66	−0.28 to 1.61	0.167	0.268
Homocitrulline	53	0.31	−0.77 to 1.39	0.573	0.764
Transsulfuration/redox module	53	0.03	−0.86 to 0.92	0.941	0.973
One-carbon/glycine module	53	−0.01	−0.63 to 0.61	0.973	0.973

Effect estimates represent standardized SGLT2i-associated coefficients from the secondary three-group model after adjustment for recalculated uncapped 2021 CKD-EPI eGFR. Benjamini–Hochberg correction was applied jointly across all eight molecular outcomes. The clinically principal comparison remains the 6-versus-10 DM-only analysis presented in Table 2. The three-group analysis does not increase the number of exposed participants and should be interpreted as secondary and exploratory. BH-FDR, Benjamini–Hochberg false-discovery rate; CI, confidence interval; Cu, copper; Mg, magnesium; Se, selenium; Zn, zinc.

## Data Availability

The data supporting the findings of this study are available from the corresponding author upon reasonable request. Public deposition of participant-level data is restricted due to ethical and privacy considerations, as the dataset contains potentially identifiable clinical information.

## Supplementary Materials

The following supporting information can be downloaded at https://www.mdpi.com/article/10.3390/nu18213510/s1, Table S1: Secondary three-group molecular estimates using recorded capped versus recalculated uncapped eGFR; Table S2: Standardized baseline differences between SGLT2i-treated and untreated participants with diabetes; Table S3: De-identified SGLT2 inhibitor exposure, concomitant medication, renal function, and standardized trace-element profiles of the six treated participants with diabetes; Table S4: Detailed small-sample analyses of serum trace-element concentrations in the DM-only cohort; Table S5: Sensitivity analysis of the SGLT2i-associated standardized serum zinc coefficient after sequential adjustment for potential confounders in the DM-only cohort; Table S6: Symmetric influence analyses for zinc and copper in the DM-only cohort; Table S7: Descriptive trace-element profiles of SGLT2 inhibitor users without diabetes; Figure S1: Individual-level serum trace-element distributions and renal-function sensitivity analyses; Figure S2: Descriptive homocitrulline phenotype and original recorded-eGFR effect estimates; Figure S3: Descriptive metabolite profiles across the three clinical phenotypes; Figure S4: Individual trace-element distributions, inter-element correlations, and a common trace-element summary; Figure S5: Descriptive trace-element profiles by dapagliflozin and empagliflozin.

## Abbreviations

The following abbreviations are used in this manuscript:

ARM64	64-bit Advanced RISC Machine architecture
Ca	Calcium
CI	confidence interval
Cu	Copper
DM	diabetes mellitus
eGFR	estimated glomerular filtration rate
FDR	false discovery rate
HbA1c	glycated hemoglobin
HC3	heteroskedasticity-consistent covariance matrix estimator (HC3)
H_2_O_2_	hydrogen peroxide
HNO_3_	nitric acid
ICP-MS	inductively coupled plasma mass spectrometry
IQR	interquartile range
K	Potassium
LC–MS/MS	liquid chromatography–tandem mass spectrometry
LVEF	left ventricular ejection fraction
Mg	Magnesium
Mo	Molybdenum
MRM	multiple-reaction monitoring
RF	Radiofrequency
Rh	Rhodium
Se	Selenium
SGLT2	sodium–glucose cotransporter 2
SGLT2i	sodium–glucose cotransporter 2 inhibitor
STROBE	Strengthening the Reporting of Observational Studies in Epidemiology
Y	Yttrium
Zn	Zinc

## References

[1] Zinman B., Wanner C., Lachin J.M., Fitchett D., Bluhmki E., Hantel S., Mattheus M., Devins T., Johansen O.E., Woerle H.J. (2015). Empagliflozin, cardiovascular outcomes, and mortality in type 2 diabetes. N. Engl. J. Med..

[2] Neal B., Perkovic V., Mahaffey K.W., de Zeeuw D., Fulcher G., Erondu N., Shaw W., Law G., Desai M., Matthews D.R. (2017). Canagliflozin and cardiovascular and renal events in type 2 diabetes. N. Engl. J. Med..

[3] Wiviott S.D., Raz I., Bonaca M.P., Mosenzon O., Kato E.T., Cahn A., Silverman M.G., Zelniker T.A., Kuder J.F., Murphy S.A. (2019). Dapagliflozin and cardiovascular outcomes in type 2 diabetes. N. Engl. J. Med..

[4] Perkovic V., Jardine M.J., Neal B., Bompoint S., Heerspink H.J.L., Charytan D.M., Edwards R., Agarwal R., Bakris G., Bull S. (2019). Canagliflozin and renal outcomes in type 2 diabetes and nephropathy. N. Engl. J. Med..

[5] McMurray J.J.V., Solomon S.D., Inzucchi S.E., Køber L., Kosiborod M.N., Martinez F.A., Ponikowski P., Sabatine M.S., Anand I.S., Bělohlávek J. (2019). Dapagliflozin in patients with heart failure and reduced ejection fraction. N. Engl. J. Med..

[6] Packer M., Anker S.D., Butler J., Filippatos G., Pocock S.J., Carson P., Januzzi J.L., Verma S., Tsutsui H., Brueckmann M. (2020). Cardiovascular and renal outcomes with empagliflozin in heart failure. N. Engl. J. Med..

[7] Heerspink H.J.L., Stefánsson B.V., Correa-Rotter R., Chertow G.M., Greene T., Hou F.F., Mann J.F.E., McMurray J.J.V., Lindberg M., Rossing P. (2020). Dapagliflozin in patients with chronic kidney disease. N. Engl. J. Med..

[8] Anker S.D., Butler J., Filippatos G., Ferreira J.P., Bocchi E., Böhm M., Brunner-La Rocca H.-P., Choi D.-J., Chopra V., Chuquiure-Valenzuela E. (2021). Empagliflozin in heart failure with a preserved ejection fraction. N. Engl. J. Med..

[9] Solomon S.D., McMurray J.J.V., Claggett B., de Boer R.A., DeMets D., Hernandez A.F., Inzucchi S.E., Kosiborod M.N., Lam C.S.P., Martinez F. (2022). Dapagliflozin in heart failure with mildly reduced or preserved ejection fraction. N. Engl. J. Med..

[10] EMPA-KIDNEY Collaborative Group (2023). Empagliflozin in patients with chronic kidney disease. N. Engl. J. Med..

[11] McGuire D.K., Shih W.J., Cosentino F., Charbonnel B., Cherney D.Z.I., Dagogo-Jack S., Pratley R., Greenberg M., Wang S., Huyck S. (2021). Association of SGLT2 inhibitors with cardiovascular and kidney outcomes in patients with type 2 diabetes: A meta-analysis. JAMA Cardiol..

[12] Heerspink H.J.L., Perkins B.A., Fitchett D.H., Husain M., Cherney D.Z.I. (2016). Sodium glucose cotransporter 2 inhibitors in the treatment of diabetes mellitus: Cardiovascular and kidney effects, potential mechanisms, and clinical applications. Circulation.

[13] Verma S., McMurray J.J.V. (2018). SGLT2 inhibitors and mechanisms of cardiovascular benefit: A state-of-the-art review. Diabetologia.

[14] Wright E.M. (2021). SGLT2 inhibitors: Physiology and pharmacology. Kidney360.

[15] Ferrannini E., Muscelli E., Frascerra S., Baldi S., Mari A., Heise T., Broedl U.C., Woerle H.J. (2014). Metabolic response to sodium-glucose cotransporter 2 inhibition in type 2 diabetic patients. J. Clin. Investig..

[16] Mulder S., Hammarstedt A., Nagaraj S.B., Nair V., Ju W., Hedberg J., Greasley P.J., Eriksson J.W., Oscarsson J., Heerspink H.J.L. (2020). A metabolomics-based molecular pathway analysis of how the sodium-glucose cotransporter-2 inhibitor dapagliflozin may slow kidney function decline in patients with diabetes. Diabetes Obes. Metab..

[17] Selvaraj S., Patel S., Sauer A.J., McGarrah R.W., Jones P., Kwee L.C., Windsor S.L., Ilkayeva O., Muehlbauer M.J., Newgard C.B. (2024). Targeted metabolomic profiling of dapagliflozin in heart failure with preserved ejection fraction: The PRESERVED-HF trial. JACC Heart Fail..

[18] Tang H., Zhang X., Zhang J., Li Y., Del Gobbo L.C., Zhai S., Song Y. (2016). Elevated serum magnesium associated with SGLT2 inhibitor use in type 2 diabetes patients: A meta-analysis of randomised controlled trials. Diabetologia.

[19] Blau J.E., Bauman V., Conway E.M., Piaggi P., Walter M.F., Wright E.C., Bernstein S., Courville A.B., Collins M.T., Rother K.I. (2018). Canagliflozin triggers the FGF23/1,25-dihydroxyvitamin D/PTH axis in healthy volunteers in a randomized crossover study. JCI Insight.

[20] Cianciolo G., De Pascalis A., Capelli I., Gasperoni L., Di Lullo L., Bellasi A., La Manna G. (2019). Mineral and electrolyte disorders with SGLT2i therapy. JBMR Plus.

[21] Liao X., Wang X., Li H., Li L., Zhang G., Yang M., Yuan L., Liu H., Yang G., Gao L. (2016). Sodium-glucose cotransporter 2 inhibitor increases circulating zinc-α2-glycoprotein levels in patients with type 2 diabetes. Sci. Rep..

[22] Maret W. (2013). Zinc biochemistry: From a single zinc enzyme to a key element of life. Adv. Nutr..

[23] Chasapis C.T., Loutsidou A.C., Spiliopoulou C.A., Stefanidou M.E. (2012). Zinc and human health: An update. Arch. Toxicol..

[24] King J.C., Brown K.H., Gibson R.S., Krebs N.F., Lowe N.M., Siekmann J.H., Raiten D.J. (2016). Biomarkers of Nutrition for Development (BOND)—Zinc review. J. Nutr..

[25] Lowe N.M., Fekete K., Decsi T. (2009). Methods of assessment of zinc status in humans: A systematic review. Am. J. Clin. Nutr..

[26] Jansen J., Karges W., Rink L. (2009). Zinc and diabetes: Clinical links and molecular mechanisms. J. Nutr. Biochem..

[27] Chabosseau P., Rutter G.A. (2016). Zinc and diabetes. Arch. Biochem. Biophys..

[28] Ranasinghe P., Pigera S., Galappatthy P., Katulanda P., Constantine G.R. (2015). Zinc and diabetes mellitus: Understanding molecular mechanisms and clinical implications. DARU J. Pharm. Sci..

[29] Wang X., Wu W., Zheng W., Fang X., Chen L., Rink L., Min J., Wang F. (2019). Zinc supplementation improves glycemic control for diabetes prevention and management: A systematic review and meta-analysis of randomized controlled trials. Am. J. Clin. Nutr..

[30] Viktorinova A., Toserova E., Krizko M., Durackova Z. (2009). Altered metabolism of copper, zinc, and magnesium is associated with increased levels of glycated hemoglobin in patients with diabetes mellitus. Metabolism.

[31] Kazi T.G., Afridi H.I., Kazi N., Jamali M.K., Arain M.B., Jalbani N., Kandhro G.A. (2008). Copper, chromium, manganese, iron, nickel, and zinc levels in biological samples of diabetes mellitus patients. Biol. Trace Elem. Res..

[32] Soinio M., Marniemi J., Laakso M., Pyörälä K., Lehto S., Rönnemaa T. (2007). Serum zinc level and coronary heart disease events in patients with type 2 diabetes. Diabetes Care.

[33] Rosenblum H., Wessler J.D., Gupta A., Maurer M.S., Bikdeli B. (2020). Zinc deficiency and heart failure: A systematic review of the current literature. J. Card. Fail..

[34] Yoshihisa A., Abe S., Kiko T., Kimishima Y., Sato Y., Watanabe S., Kanno Y., Miyata-Tatsumi M., Misaka T., Sato T. (2018). Association of serum zinc level with prognosis in patients with heart failure. J. Card. Fail..

[35] Ozyildirim S., Baltaci S.B. (2023). Cardiovascular diseases and zinc. Biol. Trace Elem. Res..

[36] Wang Z., Nicholls S.J., Rodriguez E.R., Kummu O., Hörkkö S., Barnard J., Reynolds W.F., Topol E.J., DiDonato J.A., Hazen S.L. (2007). Protein carbamylation links inflammation, smoking, uremia and atherogenesis. Nat. Med..

[37] Koeth R.A., Kalantar-Zadeh K., Wang Z., Fu X., Tang W.H.W., Hazen S.L. (2013). Protein carbamylation predicts mortality in ESRD. J. Am. Soc. Nephrol..

[38] Berg A.H., Drechsler C., Wenger J., Buccafusca R., Hod T., Kalim S., Ramma W., Parikh S.M., Steen H., Friedman D.J. (2013). Carbamylation of serum albumin as a risk factor for mortality in patients with kidney failure. Sci. Transl. Med..

[39] Jaisson S., Pietrement C., Gillery P. (2011). Carbamylation-derived products: Bioactive compounds and potential biomarkers in chronic renal failure and atherosclerosis. Clin. Chem..

[40] Tang W.H.W., Shrestha K., Wang Z., Borowski A.G., Troughton R.W., Klein A.L., Hazen S.L. (2013). Protein carbamylation in chronic systolic heart failure: Relation to renal impairment and adverse long-term outcomes. J. Card. Fail..

[41] Gorisse L., Pietrement C., Vuiblet V., Schmelzer C.E.H., Köhler M., Duca L., Debelle L., Fornès P., Jaisson S., Gillery P. (2016). Protein carbamylation is a hallmark of aging. Proc. Natl. Acad. Sci. USA.

[42] von Elm E., Altman D.G., Egger M., Pocock S.J., Gøtzsche P.C., Vandenbroucke J.P., STROBE Initiative (2007). The Strengthening the Reporting of Observational Studies in Epidemiology (STROBE) statement: Guidelines for reporting observational studies. Lancet.

[43] American Diabetes Association Professional Practice Committee for Diabetes (2026). 2. Diagnosis and classification of diabetes: Standards of Care in Diabetes—2026. Diabetes Care.

[44] Inker L.A., Eneanya N.D., Coresh J., Tighiouart H., Wang D., Sang Y., Crews D.C., Doria A., Estrella M.M., Froissart M. (2021). New creatinine- and cystatin C-based equations to estimate GFR without race. N. Engl. J. Med..

[45] Benjamini Y., Hochberg Y. (1995). Controlling the false discovery rate: A practical and powerful approach to multiple testing. J. R. Stat. Soc. Ser. B Methodol..

[46] MacKinnon J.G., White H. (1985). Some heteroskedasticity-consistent covariance matrix estimators with improved finite sample properties. J. Econom..

