# Supplementary material for: Lower Circulating Trace-Element Concentrations Among SGLT2 Inhibitor Users with Diabetes: An Exploratory Cross-Sectional Study
Source: Nutrients. 2026 Oct 25;18(21):3510. doi: 10.3390/nu18213510 (PMC13764864; doi:10.3390/nu18213510)
Supplement: Supplementary file 1 [file nutrients-18-03510-s001.zip › nutrients-4564144-supplementary.pdf]

nutrients MDPI

Supplementary Materials

Supplementary Table S1.

Secondary three-group molecular estimates using recorded capped versus recalculated uncapped eGFR.

Outcome	Renal adjustment	n	β	HC3 SE	95% CI	P	BH-FDR q
Cu	Recorded capped eGFR	51	−1.426	0.536	−2.476 to −0.376	0.008	0.061
Mg	Recorded capped eGFR	51	−1.220	0.639	−2.473 to 0.033	0.056	0.113
Se	Recorded capped eGFR	51	−1.135	0.468	−2.053 to −0.217	0.015	0.061
Zn	Recorded capped eGFR	51	−0.726	0.369	−1.450 to −0.003	0.049	0.113
Homocitrulline	Recorded capped eGFR	51	0.505	0.411	−0.300 to 1.311	0.219	0.292
Lysine/carbamylation module	Recorded capped eGFR	51	0.741	0.450	−0.142 to 1.624	0.100	0.160
One-carbon/glycine module	Recorded capped eGFR	51	−0.105	0.280	−0.653 to 0.444	0.708	0.760
Transsulfuration/redox module	Recorded capped eGFR	51	0.147	0.483	−0.800 to 1.094	0.760	0.760
Cu	Recalculated uncapped 2021 CKD-EPI eGFR	53	−1.522	0.490	−2.483 to −0.561	0.002	0.015
Mg	Recalculated uncapped 2021 CKD-EPI eGFR	53	−1.335	0.592	−2.495 to −0.175	0.024	0.051
Se	Recalculated uncapped 2021 CKD-EPI eGFR	53	−1.145	0.455	−2.037 to −0.253	0.012	0.047
Zn	Recalculated uncapped 2021 CKD-EPI eGFR	53	−0.732	0.328	−1.374 to −0.090	0.026	0.051
Homocitrulline	Recalculated uncapped 2021 CKD-EPI eGFR	53	0.312	0.553	−0.772 to 1.395	0.573	0.764
Lysine/carbamylation module	Recalculated uncapped 2021 CKD-EPI eGFR	53	0.664	0.481	−0.278 to 1.606	0.167	0.268
One-carbon/glycine module	Recalculated uncapped 2021 CKD-EPI eGFR	53	−0.011	0.315	−0.629 to 0.607	0.973	0.973

Footnote: Effect estimates represent the SGLT2i-associated coefficient from secondary three-group regression models using participants with diabetes not receiving an SGLT2 inhibitor as the reference group and including an indicator for participants without diabetes. Each model was adjusted either for the clinically recorded eGFR, with values ≥60 mL/min/1.73 m² capped at 60 mL/min/1.73 m², or for eGFR recalculated using the uncapped 2021 CKD-EPI creatinine equation. Molecular outcomes were expressed on standardized or prespecified module-score scales. HC3 heteroskedasticity-consistent standard errors were used, and 95% confidence intervals were calculated as β ± 1.96 × HC3 SE. Benjamini–Hochberg false-discovery-rate correction was applied jointly across the eight molecular outcomes within each renal-function analysis. Negative coefficients indicate lower molecular values among participants with diabetes receiving SGLT2i compared with those not receiving SGLT2i. The recorded-eGFR analysis included 51 participants because two participants lacked the corresponding clinical eGFR value; the recalculated uncapped-eGFR analysis included 53 participants. These secondary analyses do not increase the number of SGLT2i-exposed participants and should be interpreted as exploratory.
Abbreviations: BH-FDR, Benjamini–Hochberg false-discovery rate; CI, confidence interval; Cu, copper; eGFR, estimated glomerular filtration rate; Mg, magnesium; Se, selenium; SGLT2i, sodium–glucose cotransporter-2 inhibitor; Zn, zinc.

Supplementary Table S2.

Standardized baseline differences between SGLT2i-treated and untreated participants with diabetes.

Characteristic	n treated	n untreated	SMD	Hedges' g*	∣SMD∣ category
MRA use	6	10	1.434	—	≥0.50
Diuretic use	6	10	1.277	—	≥0.50
ACE inhibitor use	6	10	−1.277	—	≥0.50
LDL cholesterol, mg/dL	6	10	−1.218	−1.152	≥0.50
Total cholesterol, mg/dL	5	10	−1.120	−1.054	≥0.50
Beta-blocker use	6	10	−0.970	—	≥0.50
HbA1c, %	5	7	0.813	0.750	≥0.50
Glucose, mg/dL	6	10	0.799	0.756	≥0.50
BMI, kg/m²	4	8	0.730	0.674	≥0.50
ARB use	6	10	0.663	—	≥0.50
HDL cholesterol, mg/dL	6	10	−0.613	−0.580	≥0.50
Recalculated uncapped eGFR, mL/min/1.73 m²	6	10	−0.590	−0.558	≥0.50
Dyslipidemia	6	10	−0.591	—	≥0.50
Triglycerides, mg/dL	6	10	0.525	0.497	≥0.50
Creatinine, mg/dL	6	10	0.520	0.491	≥0.50
Coronary artery disease/IHD	6	10	0.471	—	0.20–0.49
Previous myocardial infarction	6	10	−0.471	—	0.20–0.49
Male sex	6	10	0.417	—	0.20–0.49
Hypertension	6	10	−0.197	—	<0.20
Atrial fibrillation	6	10	0.197	—	<0.20
Chronic kidney disease	6	10	0.197	—	<0.20
Statin use	6	10	−0.197	—	<0.20
Age, years	6	10	0.186	0.176	<0.20
LVEF, %	6	10	0.100	0.094	<0.20

Footnote: Standardized mean differences (SMDs) compare participants with diabetes receiving an SGLT2 inhibitor (treated) with participants with diabetes not receiving an SGLT2 inhibitor (untreated); positive values indicate higher values or greater prevalence in the treated group. For continuous variables, SMDs were calculated using the pooled standard deviation, and Hedges' g applies the corresponding small-sample bias correction. For binary variables, standardized differences in proportions are reported and Hedges' g is not applicable. Absolute SMDs were categorized descriptively as <0.20, 0.20–0.49, or ≥0.50. These thresholds are used to characterize covariate imbalance rather than statistical significance. Sample sizes vary for variables with missing observations. ACE, angiotensin-converting enzyme; ARB, angiotensin receptor blocker; BMI, body mass index; DM, diabetes mellitus; eGFR, estimated glomerular filtration rate; HbA1c, glycated hemoglobin; HDL, high-density lipoprotein; IHD, ischemic heart disease; LDL, low-density lipoprotein; LVEF, left ventricular ejection fraction; MRA, mineralocorticoid receptor antagonist; SGLT2i, sodium–glucose cotransporter-2 inhibitor.

Supplementary Table S3.

De-identified SGLT2 inhibitor exposure, concomitant medication, renal function, and standardized trace-element profiles of the six treated participants with diabetes.

Participant	SGLT2i agent	Dose	ACEi	ARB	ARNI	β-blocker	MRA	Diuretic	Statin	Recorded eGFR	Uncapped eGFR	Cu z score	Mg z score	Se z score	Zn z score
T1	Dapagliflozin	10 mg	1	0	0	1	1	1	1	47.0	49.8	−1.59	−1.85	−1.92	−1.27
T2	Empagliflozin	10 mg	0	1	0	1	1	1	1	54.0	58.8	−1.77	−1.64	−1.39	−1.16
T3	Empagliflozin	10 mg	0	1	0	1	0	0	1	60.0	85.5	−1.19	−2.35	−1.62	−1.00
T4	Dapagliflozin	10 mg	0	1	0	0	1	1	1	60.0	85.3	0.01	−0.16	−0.01	0.13
T5	Dapagliflozin	10 mg	0	0	0	0	1	1	1	49.0	53.0	−0.27	0.75	0.01	−0.48
T6	Empagliflozin	10 mg	0	0	0	0	0	1	0	60.0	95.1	−1.64	−2.00	−1.61	−0.91

Footnote: All six participants received an SGLT2 inhibitor at a dose of 10 mg daily; treatment duration and adherence were unavailable. Trace-element values are expressed as z scores calculated from the final serum-equivalent concentrations within the 53-participant cohort included in the formal three-group analyses. Positive values indicate concentrations above the corresponding cohort mean, and negative values indicate concentrations below it. Participant labels (T1–T6) are de-identified and are used solely to display individual-level patterns. Medication variables are coded as 1 = present and 0 = absent.
Abbreviations: ACEi, angiotensin-converting enzyme inhibitor; ARB, angiotensin receptor blocker; ARNI, angiotensin receptor–neprilysin inhibitor; Cu, copper; eGFR, estimated glomerular filtration rate; Mg, magnesium; MRA, mineralocorticoid receptor antagonist; Se, selenium; SGLT2i, sodium–glucose cotransporter-2 inhibitor; Zn, zinc.

Supplementary Table S4.

Detailed small-sample analyses of serum trace-element concentrations in the DM-only cohort.

Element	Treated mean (μg/L)	Untreated mean (μg/L)	Mean difference	Exact P	Rank-biserial	Permutation P	Adjusted β	95% CI	P
Cu	685.7	1043.4	−357.7	0.016	−0.733	0.014	−1.44	−2.36 to −0.53	0.002
Mg	13421.7	18316.1	−4894.4	0.118	−0.500	0.042	−1.34	−2.65 to −0.03	0.045
Se	60.0	90.2	−30.2	0.022	−0.700	0.013	−1.23	−2.20 to −0.26	0.013
Zn	503.6	835.9	−332.3	0.031	−0.667	0.038	−0.78	−1.44 to −0.13	0.019

Footnote: Analyses compare six participants with diabetes receiving an SGLT2 inhibitor with ten participants with diabetes not receiving an SGLT2 inhibitor. Exact two-sided Mann–Whitney tests and rank-biserial effect sizes were used as distribution-free comparisons. Exhaustive permutation testing evaluated all 8008 possible allocations of six exposed participants among the 16 participants with diabetes. Permutation P values were calculated for the observed between-group difference in mean serum concentration. Renal-adjusted coefficients were obtained from regression models including prevalent SGLT2i exposure and recalculated uncapped 2021 CKD-EPI eGFR, with HC3 heteroskedasticity-consistent standard errors. Negative mean differences, rank-biserial coefficients, and regression coefficients indicate lower concentrations among SGLT2i users. These complementary analyses interrogate the same observations and should not be interpreted as independent replication.
Abbreviations: CI, confidence interval; CKD-EPI, Chronic Kidney Disease Epidemiology Collaboration; Cu, copper; DM, diabetes mellitus; eGFR, estimated glomerular filtration rate; Mg, magnesium; Se, selenium; SGLT2i, sodium–glucose cotransporter-2 inhibitor; Zn, zinc.

Supplementary Table S5.

Sensitivity analysis of the SGLT2i-associated standardized serum zinc coefficient after sequential adjustment for potential confounders in the DM-only cohort.

Model	n	SGLT2i-associated β	HC3 SE	95% CI	P value
Uncapped eGFR-adjusted reference model	16	−0.782	0.333	−1.435 to −0.129	0.019
Uncapped eGFR + BMI	12	−1.210	0.180	−1.563 to −0.856	<0.001
Uncapped eGFR + HbA1c	12	−0.746	0.595	−1.912 to 0.420	0.210
Uncapped eGFR + diuretic use	16	−0.800	0.508	−1.795 to 0.196	0.116
Uncapped eGFR + MRA use	16	−0.922	0.245	−1.402 to −0.441	<0.001

Footnote: Effect estimates represent the SGLT2i-associated coefficient for standardized serum zinc concentration among participants with diabetes receiving an SGLT2 inhibitor compared with participants with diabetes not receiving an SGLT2 inhibitor. The reference model included prevalent

SGLT2i exposure and recalculated uncapped 2021 CKD-EPI eGFR. BMI, HbA1c, diuretic use, and MRA use were subsequently added individually to the reference model. HC3 heteroskedasticity-consistent standard errors were used throughout, and 95% confidence intervals were calculated as β ± 1.96 × HC3 SE. No covariates were imputed; therefore, the BMI- and HbA1c-adjusted models were restricted to 12 complete cases. Given the small sample size and the reduced complete-case samples for BMI and HbA1c, these models were performed solely to assess the direction and magnitude of the SGLT2i-associated zinc coefficient under alternative model specifications and should not be interpreted as independently adjusted treatment-effect estimates.
Abbreviations: BMI, body mass index; CI, confidence interval; CKD-EPI, Chronic Kidney Disease Epidemiology Collaboration; eGFR, estimated glomerular filtration rate; HbA1c, glycated hemoglobin; MRA, mineralocorticoid receptor antagonist; SGLT2i, sodium–glucose cotransporter-2 inhibitor; Zn, zinc; β, regression coefficient.

Supplementary Table S6.

Symmetric influence analyses for zinc and copper in the DM-only cohort.

Element	Full-model β	Full-model 95% CI	Full-model P	No. of LOO models	Range of LOO β	Direction retained	Range of LOO P	Maximum Cook's D	Maximum absolute exposure DFBETA
Zn	−0.782	−1.435 to −0.129	0.019	16	−1.014 to −0.683	16/16 negative	<0.001–0.079	0.251	0.744
Cu	−1.442	−2.356 to −0.528	0.002	16	−1.716 to −1.306	16/16 negative	<0.001–0.012	0.498	0.547

Footnote: The full renal-adjusted models compared six participants with diabetes receiving an SGLT2 inhibitor with ten participants with diabetes not receiving an SGLT2 inhibitor and included recalculated uncapped 2021 CKD-EPI eGFR as the renal covariate. Leave-one-observation-out (LOO) analyses sequentially omitted each of the 16 participants and refitted the corresponding renal-adjusted model. Cook's distance and DFBETA values were obtained from the full regression models and are reported as the maximum observed value across participants. The SGLT2i-associated coefficient remained negative in all 16 LOO iterations for both Zn and Cu. These analyses assess sensitivity to individual observations but do not constitute independent replication, demonstrate robustness to residual confounding, or establish a causal treatment effect.
Abbreviations: CI, confidence interval; Cu, copper; eGFR, estimated glomerular filtration rate; LOO, leave-one-observation-out; SGLT2i, sodium–glucose cotransporter-2 inhibitor; Zn, zinc; β, regression coefficient.

Supplementary Table S7.

Descriptive trace-element profiles of SGLT2 inhibitor users without diabetes.

Participant	Recorded SGLT2i	Dose	Cu (μg/L)	Mg (μg/L)	Se (μg/L)	Zn (μg/L)
ND1	Diaflix	10 mg	1134.7	21,199.5	106.7	1105.3
ND2	Jardiance	10 mg	1298.3	22,379.1	132.1	1255.7
ND3	Forxiga	10 mg	899.6	20,872.8	87.1	1085.5
Observed range	—	—	899.6–1298.3	20,872.8–22,379.1	87.1–132.1	1085.5–1255.7

Footnote: The three participants receiving an SGLT2 inhibitor in the absence of diabetes were excluded from all formal treatment-inference models and are presented here solely for descriptive context. Trace-element concentrations are serum-equivalent values derived using the final analytical processing applied throughout the study. Because only three such participants were available, no formal statistical comparison was performed and these observations should not be interpreted as evidence of effect modification by diabetes status. Participant labels ND1–ND3 are de-identified.
Abbreviations: Cu, copper; DM, diabetes mellitus; Mg, magnesium; Se, selenium; SGLT2i, sodium–glucose cotransporter-2 inhibitor; Zn, zinc.

Supplementary Figure S1. Individual-level serum trace-element distributions and renal-function sensitivity analyses.

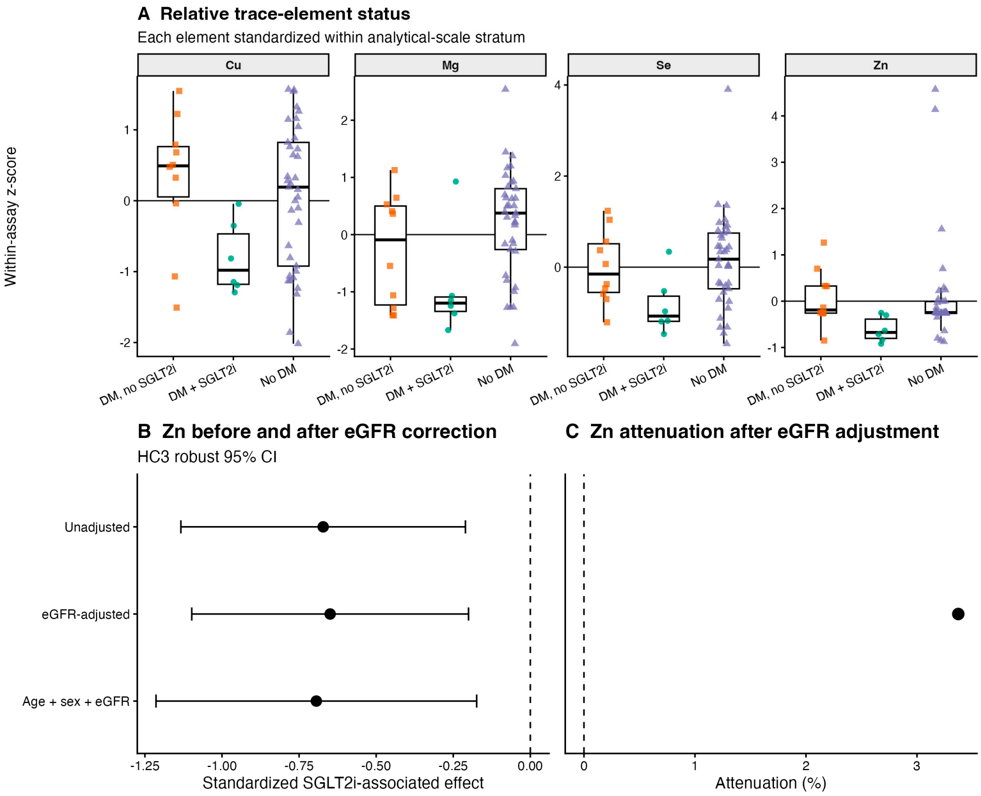

Abbreviations: CI, confidence interval; CKD-EPI, Chronic Kidney Disease Epidemiology Collaboration; Cu, copper; DM, diabetes mellitus; eGFR, estimated glomerular filtration rate; HC3, heteroskedasticity-consistent covariance estimator type 3; Mg, magnesium; Se, selenium; SGLT2i, sodium–glucose cotransporter-2 inhibitor; Zn, zinc.

Supplementary Figure S2. Descriptive homocitrulline phenotype and original recorded-eGFR effect estimates. The revised principal uncapped-eGFR analysis attenuated the homocitrulline association and did not support statistical significance.

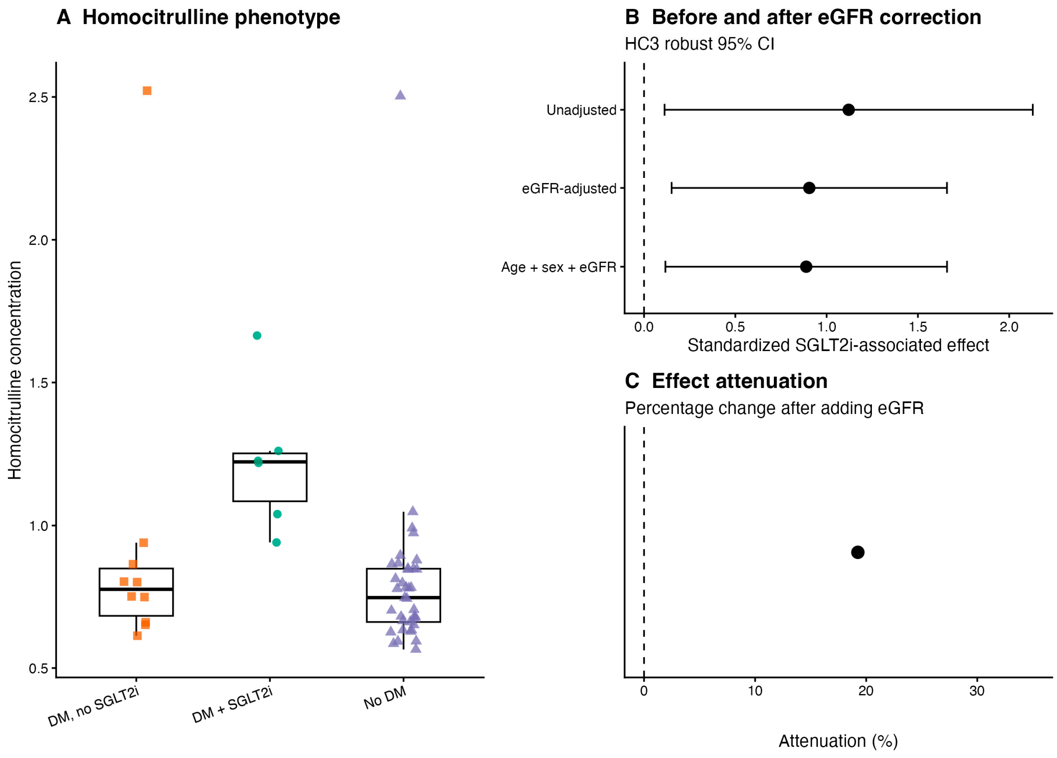

Abbreviations: CI, confidence interval; DM, diabetes mellitus; eGFR, estimated glomerular filtration rate; HC3, heteroskedasticity-consistent covariance estimator type 3; SGLT2i, sodium–glucose cotransporter-2 inhibitor.

Supplementary Figure S3. Descriptive metabolite profiles across the three clinical phenotypes. Cells represent group-median standardized concentrations; no individual cell should be interpreted as inferentially significant.

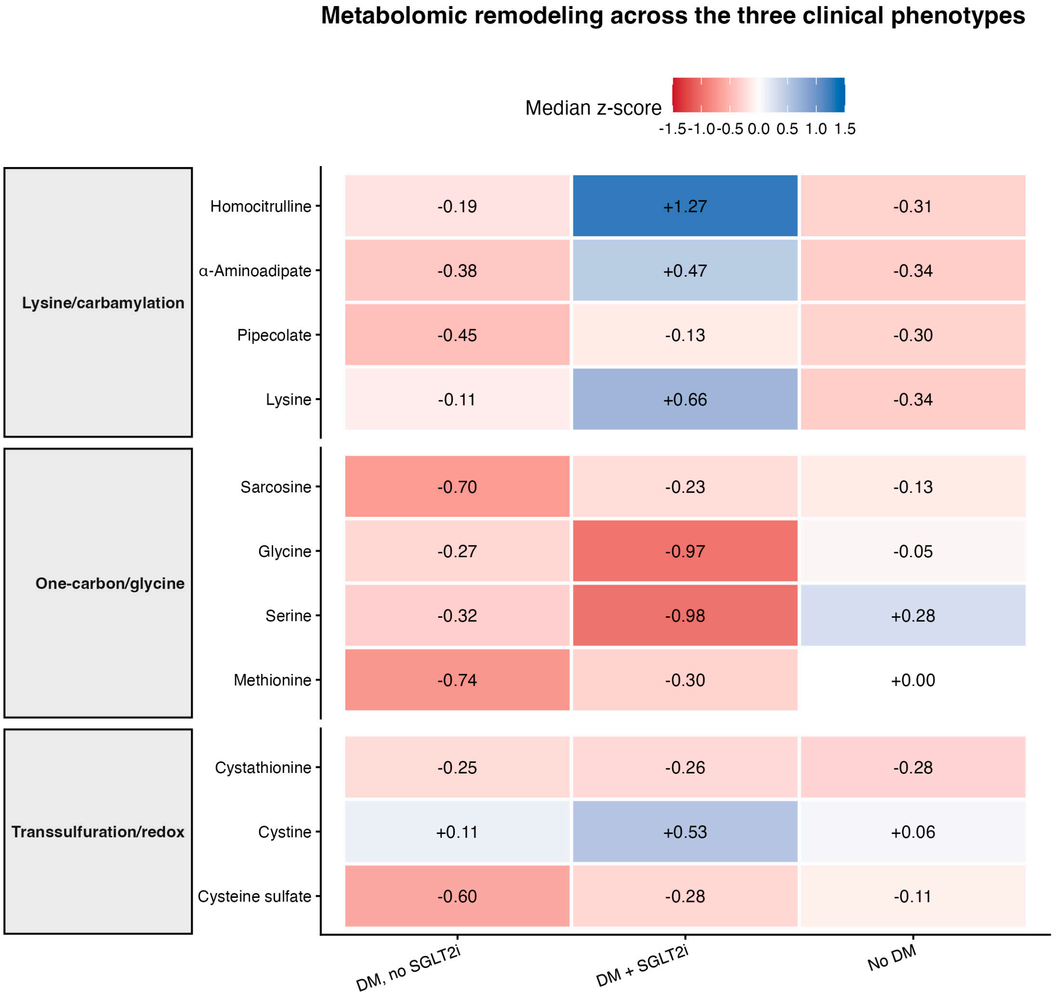

Abbreviations: DM, diabetes mellitus; SGLT2i, sodium–glucose cotransporter-2 inhibitor.

Supplementary Figure S4.

Individual trace-element distributions, inter-element correlations, and a common trace-element summary.

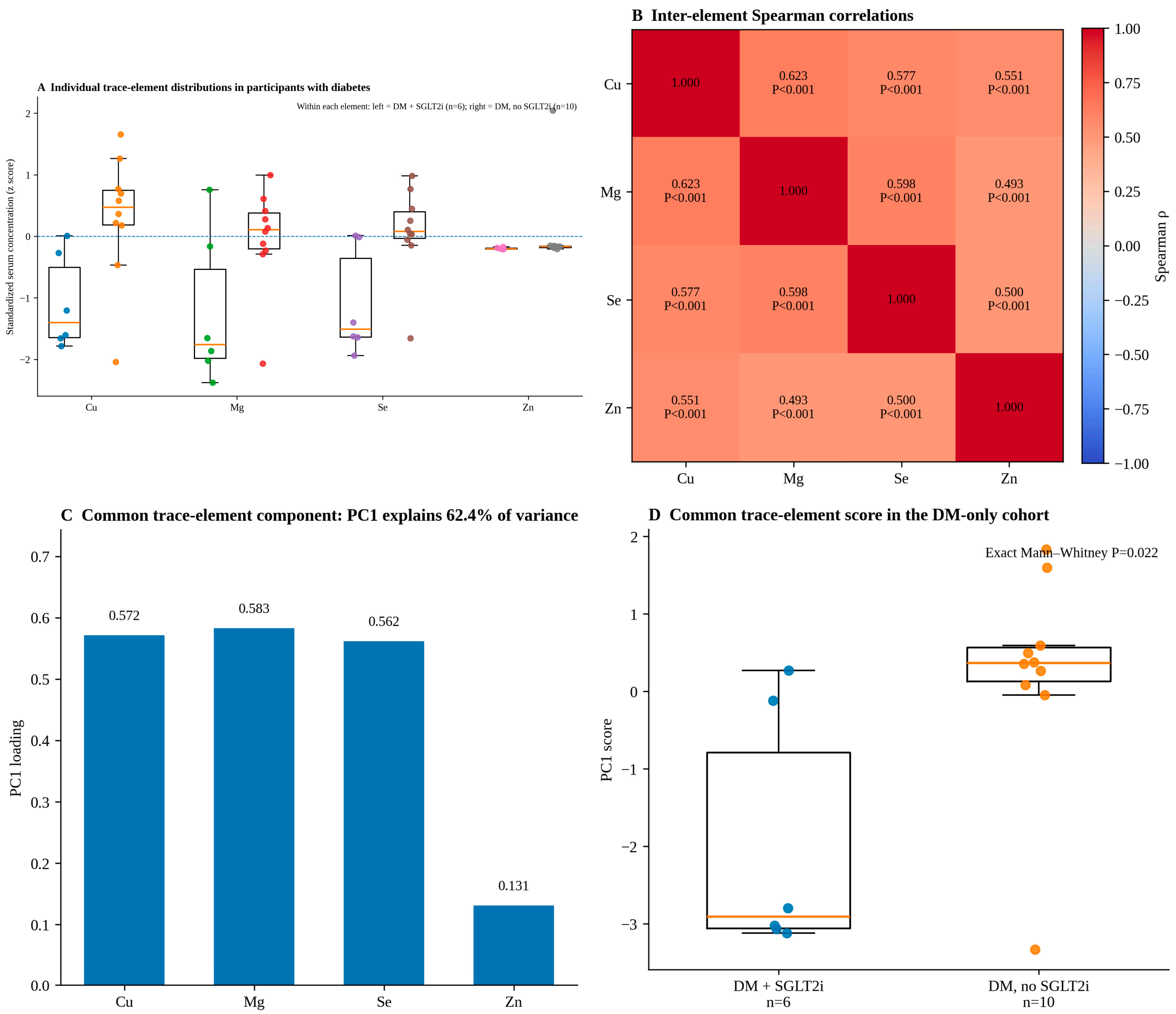

(A) Individual standardized serum concentrations of copper (Cu), magnesium (Mg), selenium (Se), and zinc (Zn) in participants with diabetes mellitus (DM), stratified according to prevalent SGLT2 inhibitor use. Individual observations are superimposed on boxplots to illustrate the distribution and between-participant variability within the small DM-only cohort (SGLT2i, $n = 6$; no SGLT2i, $n = 10$).
(B) Spearman correlation matrix for the four serum trace elements in the formal analysis cohort. Values within the matrix represent Spearman correlation coefficients (ϱ), with corresponding P values. The uniformly positive correlations indicate a shared covariance structure across the measured elements.
(C) Loadings of Cu, Mg, Se, and Zn on the first principal component (PC1), derived from standardized trace-element concentrations. Concordant positive loadings indicate that PC1 represents a common multielement dimension rather than variation driven by a single trace element. The proportion of total trace-element variance explained by PC1 is indicated in the panel.
(D) Distribution of PC1 scores in the DM-only cohort according to prevalent SGLT2i exposure. Higher PC1 scores represent a generally higher circulating trace-element profile. Individual observations are shown together with boxplots; the displayed P value derives from an exact two-sided Mann–Whitney test.
Footnote: Trace-element concentrations were expressed as serum-equivalent values and standardized before multivariate analysis. Panels A and D display individual observations to preserve transparency regarding the small exposed subgroup. The correlation and principal-component analyses were exploratory and were undertaken to characterize the shared structure of the four trace elements; they should not be interpreted as independent confirmation of the element-specific associations or as evidence of a causal effect of SGLT2 inhibition. Boxplots show the median and interquartile range, with whiskers extending according to the conventional 1.5 × IQR criterion.
Abbreviations: Cu, copper; DM, diabetes mellitus; IQR, interquartile range; Mg, magnesium; PC1, first principal component; Se, selenium; SGLT2i, sodium–glucose cotransporter-2 inhibitor; Zn, zinc.

Supplementary Figure S5.

Descriptive trace-element profiles by dapagliflozin and empagliflozin.

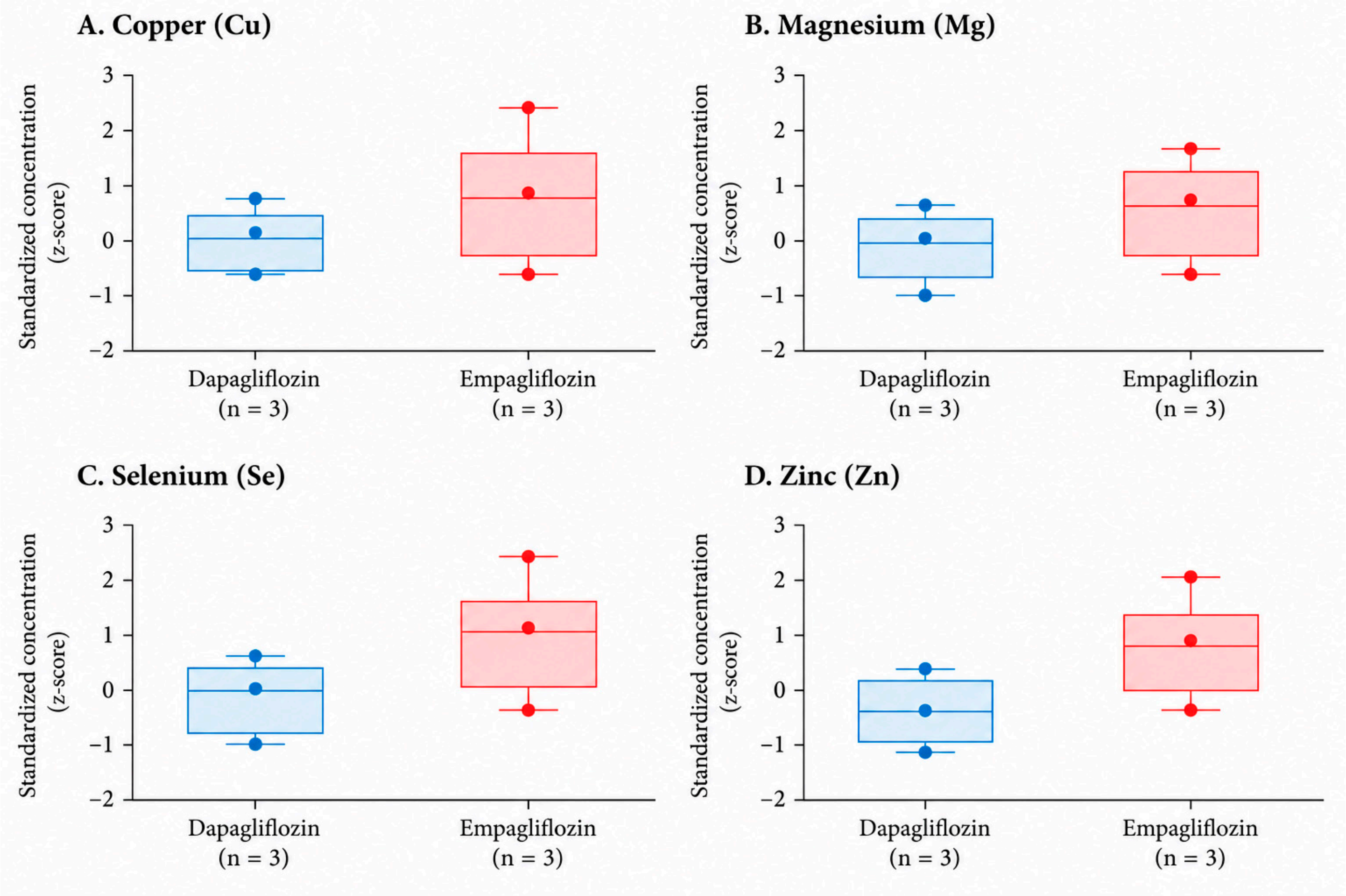

(A–D) Individual standardized serum concentrations of (A) copper, (B) magnesium, (C) selenium, and (D) zinc among the six participants with diabetes receiving an SGLT2 inhibitor, stratified descriptively according to the recorded SGLT2 inhibitor. Three participants received dapagliflozin and three received empagliflozin. Trace-element concentrations are presented as standardized values (*z* scores) to facilitate visualization of the relative multielement profiles across agents. Individual observations are superimposed on boxplots to preserve transparency regarding the very small subgroup sizes and the underlying between-participant variability. Boxplots indicate the median and interquartile range, with whiskers extending according to the conventional 1.5 × IQR criterion. These agent-specific distributions are presented for descriptive purposes only. Given the extremely small sample size ($n = 3$ per agent), no formal between-agent statistical testing was performed, and the apparent differences between dapagliflozin and empagliflozin should not be interpreted as evidence of agent-specific effects. In particular, the figure does not establish differential effects of the two SGLT2 inhibitors on trace-element concentrations and should be regarded as an individual-level visualization complementary to the pooled SGLT2i analysis.

Abbreviations: Cu, copper; DM, diabetes mellitus; IQR, interquartile range; Mg, magnesium; Se, selenium; SGLT2i, sodium–glucose cotransporter-2 inhibitor; Zn, zinc.
